# Successful peritoneal dialysis using a percutaneous tube for peritoneal drainage in an extremely low birth weight infant: a case report

**DOI:** 10.1186/s40792-017-0390-3

**Published:** 2017-11-09

**Authors:** Satoshi Yokoyama, Takayuki Nukada, Yuka Ikeda, Shigeto Hara, Akira Yoshida

**Affiliations:** 1 Department of Pediatric Surgery, Japanese Red Cross Society, Wakayama Medical Center, 4-20 Komatsubara-dori, Wakayama, Wakayama 640-8558 Japan; 2 Department of Pediatrics, Japanese Red Cross Society, Wakayama Medical Center, 4-20 Komatsubara-dori, Wakayama, Wakayama 640-8558 Japan

**Keywords:** Peritoneal dialysis, Acute renal injury, Extremely low birth weight infant

## Abstract

**Background:**

Peritoneal dialysis (PD) for acute kidney injury (AKI) of newborns has been performed safely. AKI occurs in 8 to 24% of extremely low birth weight (ELBW) infants. Although PD has only been used occasionally in ELBW infants, prognosis is poor for ELBW infants with AKI. Several reports have described successful PD in these infants, but no guideline-based evidence concerning indications for renal replacement therapy in ELBW infants are currently available. Here, we report on our experience with PD in an ELBW infant with AKI resulting from septic shock.

**Case presentation:**

A male was born at 24 weeks and 3 days gestation weighing 264 g by emergency cesarean section due to complications of pregnancy in a patient with hemolysis, elevated liver enzymes, low platelets (HELLP) syndrome. On day of life (DOL) 15, the inability to ventilate, along with cardiovascular dysfunction, acute kidney injury, and ascites under tension led to the tentative diagnosis of abdominal compartment syndrome (ACS). On DOL 17, placement of a percutaneous drainage tube immediately released compression of the tense abdomen. Although intra-abdominal pressure reduction with percutaneous drainage temporarily improved respiratory status, circulatory impairment persisted and infections were not well controlled. Finally, the patient developed anuria. On DOL 21, peritoneal dialysis (PD) was started by initially inserting a drainage tube. Although the patient had catheter-associated peritonitis, urine output improved by DOL 44 and PD was discontinued on DOL 53. On DOL 75, extubation was conducted without circulatory dysfunction. The patient was discharged on DOL 224.

**Conclusions:**

We emphasize that starting PD treatment before the onset of anuria is important in ELBW infants with AKI. Although the catheter used in our case was initially inserted for drainage of ascites, this type of catheter is sufficiently useful for PD in ELBW infants, and PD using a drainage tube may represent a safe, effective, and minimally invasive treatment for ELBW infants. To our knowledge, this is the first report to describe the use of a percutaneous tube to conduct successful PD for peritoneal drainage in an ELBW infant. This is the lowest-weight ELBW infant with successful PD reported to date.

## Background

Peritoneal dialysis (PD) for acute kidney injury (AKI) of newborns has been performed safely. AKI occurs in 8 to 24% of extremely low birth weight (ELBW) infants, and although PD has only occasionally been conducted in ELBW infants, prognosis is poor for ELBW infants with AKI. Moreover, dialysis catheters suitable for ELBW infants are difficult to find due to their small body size and inelastic abdominal wall. Although several reports have described successful PD, no guideline-based evidence concerning indications for renal replacement therapy in ELBW infants has appeared. We report here on our experience with PD in an ELBW infant with AKI resulting from septic shock.

## Case presentation

**Fig. 1:**
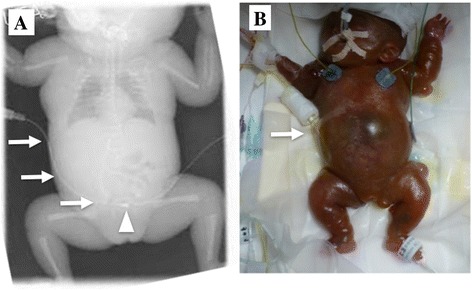
**a** Abdominal X-ray of the patient on DOL 17. Arrows indicate the percutaneous drainage tube. The tip of the catheter was placed in the Douglas pouch (arrow head). **b** Photograph of the patient on DOL 21. PD was started by initially inserting a drainage tube. The arrow indicates the initially inserted drainage tube for peritoneal drainage

A male was born at 24 weeks and 3 days gestation weighing 264 g by emergency cesarean section due to complications of pregnancy in a patient with hemolysis, elevated liver enzymes, low platelets (HELLP) syndrome. Apgar score was 4 at 1 min and 6 at 5 min after birth. On day of life (DOL) 3, the patient had massive pulmonary hemorrhage and severe intraventricular hemorrhage resulting from reopening of the ductus arteriosus. The comorbid sepsis required antibiotic therapy and multiple hemotransfusions. On DOL 5, the patient became progressively hypoxemic and hypotensive with worsening lactic acidosis refractory to massive fluid resuscitation and inotropic support, suggesting fungal septic shock. On DOL 15, as a result of ongoing capillary leak syndrome, generalized edema, increasing abdominal distension, and ascetic fluid accumulation were noted. The inability to ventilate, along with cardiovascular dysfunction, AKI, and ascites under tension led to a tentative diagnosis of abdominal compartment syndrome (ACS). On DOL 17, a pediatric surgeon inserted a percutaneous drainage tube (6 Fr single-lumen, Trocar Aspiration Kit®, Covidien Japan Inc., Tokyo, Japan) into the peritoneal cavity in the right part of the umbilical region under echographic guidance at the bedside (Fig. 1a). Over 20 ml of clear ascitic fluid was immediately drained, and compression of the tense abdomen was released. Although intra-abdominal pressure reduction with percutaneous drainage temporarily improved respiratory status, circulatory impairment persisted and infections were not well controlled. Finally, the patient developed anuria. Accompanying electrolyte abnormalities included raised serum blood urea nitrogen (BUN) (44 mg/dl) and serum creatinine levels (1.54 mg/dl). Blood lactic acid levels remained elevated (between 64 and 84 mg/dl). On DOL 21, peritoneal dialysis (PD) was started by initially inserting a drainage tube (Fig. 1b). PD was started using a dialysis solution of 1.5% glucose; a small fill volume of 3 ml (10 ml/kg) of dialysis solution, which was gradually increased to 30 ml/kg; a dwell time of 90 min; drainage time of 75 min; and 8 cycles per day. Leakage of peritoneal fluid from the exit site was noted, which did not impair PD efficacy, and was effectively stopped by application of 2-octylcyanoacrylate (Dermabond®; Ethicon, Inc., Somerville, NJ) around the exit site of the tube (Fig. 3). Although the patient had catheter-associated peritonitis, urine output had improved by DOL 44, and PD was discontinued on DOL 53 (834 g bodyweight). On DOL 75 (589 g bodyweight), extubation was conducted without circulatory dysfunction. The patient was discharged on DOL 224. He relied on home oxygen therapy and nasogastric tube feeding due to swallowing difficulties and gastroesophageal reflux. During the 12-month follow-up, cerebellar atrophy was demonstrated on magnetic resonance imaging (MRI); however, there was no indication of severe neurological developmental disabilities. The patient requires careful ongoing follow-up.

## Discussion

This case has two clinical implications. The first is that starting PD treatment before the onset of anuria is important in ELBW infants with AKI. The second is that although the catheter used in our case was initially inserted for drainage of ascites, this type of catheter is sufficiently useful for PD in ELBW infants, and PD using a drainage tube may represent a safe, effective, and minimally invasive treatment for ELBW infants.

ACS is underreported in children, and the actual incidence in critically ill patients may be much higher than the reported incidence of 0.9 to 12%. In patients with established ACS, the reported mortality rate is between 50 and 80% [1]. ACS is categorized as “primary” if it is related to injury or disease of the abdominopelvic region (trauma, aneurysm rupture, hemoperitoneum, acute pancreatitis, retroperitoneal bleeding) and “secondary” if it is related to systemic conditions (sepsis, major burns, capillary leak, massive fluid resuscitation). ACS in ELBW infants is demonstrated clinically by worsening respiratory ventilation, increased abdominal circumference, and oliguria. However, currently accepted monitoring techniques cannot be used in ELBW infants. The earliest sign of ACS in ELBW infants is the inability to ventilate accompanied by increased abdominal girth, occurring well before anuria. However, the three signs of ACS are almost uniformly present, albeit to varying degrees, in all septic ELBW infants. Therefore, a possible diagnosis of ACS should be considered much more frequently than it generally is.

Timely placement of a percutaneous drainage tube in the abdominal cavity is effective and less invasive and can be deployed successfully for ELBW infants with ACS [2]. In our case, we observed a temporary improvement in respiratory status and circulatory impairment after decompression by ascetic fluid drainage using a percutaneous drainage tube. However, the patient developed anuria. We hypothesize that AKI may have been associated with the onset of neonatal sepsis, the immaturity of organs associated with ELBW, a severe form respiratory distress syndrome that requires high parameters of mechanical ventilation, or hypotension. We further emphasize that starting PD treatment before the onset of anuria is important in ELBW infants with AKI.

**Fig. 2:**
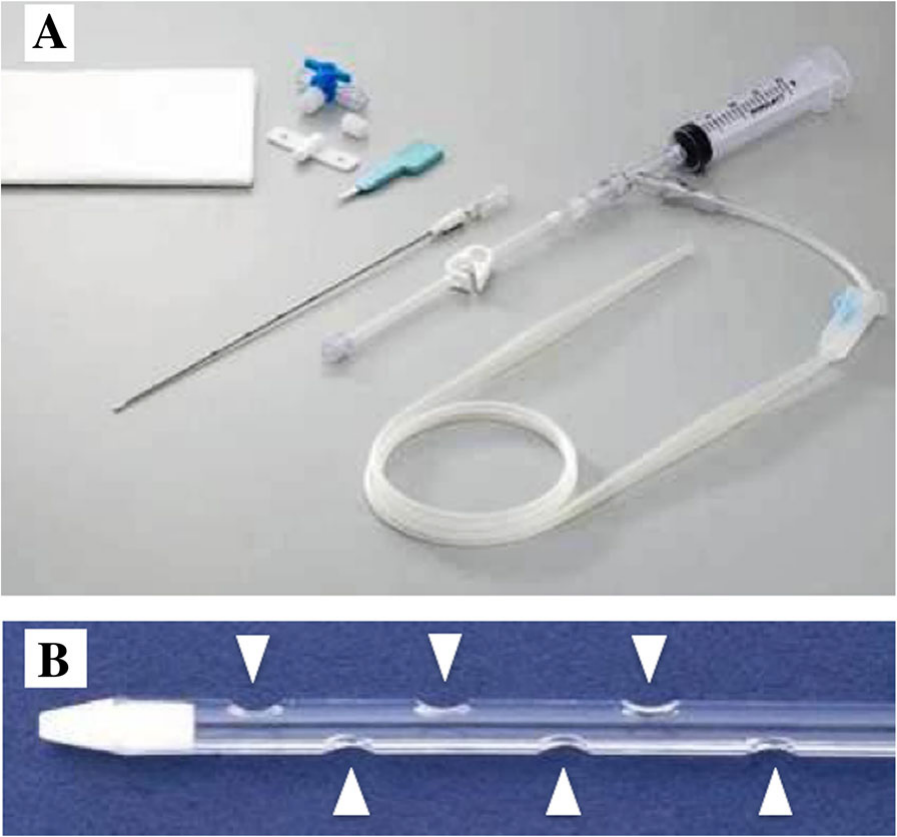
**a** The drainage tube was placed by direct puncture using a needle, making its use minimally invasive, safe and minimally traumatic. **b** Unlike intravenous cannulas used for PD in ELBW infants, this catheter is made of soft polyurethane for kink resistance and shape memory and has lateral channels at its end (arrow heads)

ELBW infants have not previously been considered eligible for PD because of technical limitations (lack of small-sized catheters and volume cyclers) and high infection rates [3–5]. Several PD access catheters have been used in small infants [6, 7]. However, these catheters have only been used for short periods and no standard method has been developed. Although the catheter used in our case was initially inserted for drainage of ascites, it was eventually used for PD. This drainage tube was placed by direct puncture using a needle, making its use minimally invasive, safe, and less traumatic (Fig. 2a). Unlike intravenous cannulas used for PD in ELBW infants, this catheter is made of soft polyurethane for kink resistance and shape memory and has lateral channels at its end, as indicated in Fig. 2b. As a consequence, it does not kink or become blocked, and in our present case, it was used for PD for an extended period of time. This suggests that the catheter is sufficiently useful for PD in ELBW infants and that PD using a drainage tube may represent a safe, effective, and minimally invasive treatment for ELBW infants.

**Fig. 3:**
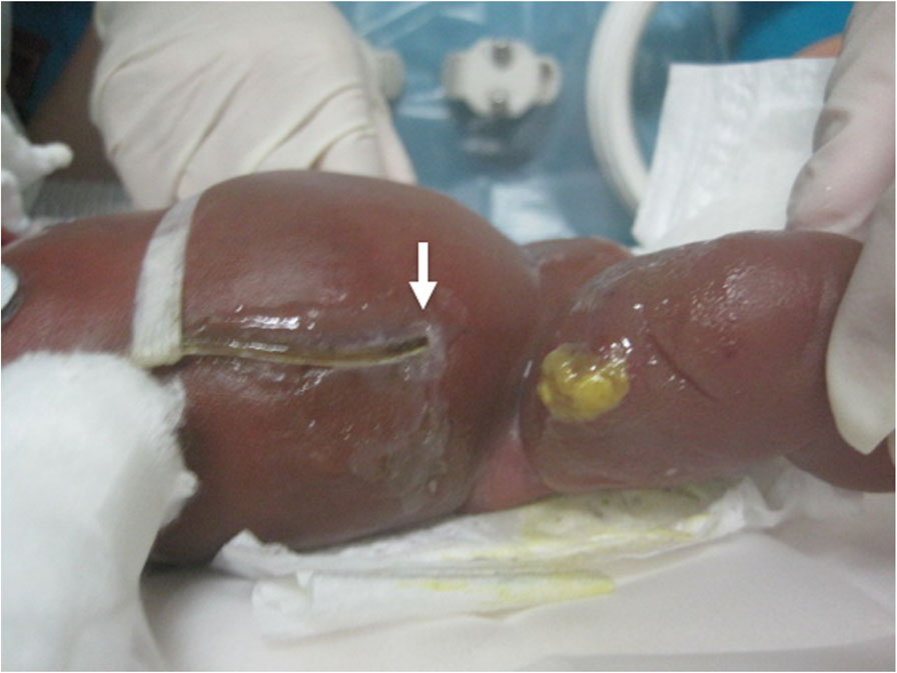
Application of 2-octylcyanoacrylate (Dermabond®, Ethicon, Inc., Somerville, NJ) around the exit site of the tube was effective for stopping pericatheter leakage in PD. The arrow indicates the site of tube insertion

Reported complications of PD in infants include peritonitis, leakage at the exit site, catheter obstruction requiring revision or replacement, and in several case reports, bowel perforation. In our case, we did experience leakage of peritoneal fluid from the exit site, which did not impair PD efficacy. Because the skin of ELBW neonates is very thin due to the lack of subcutaneous fat tissue, firm fixation of the PD tube to the abdominal wall is difficult. In our case, pericatheter leakage was observed at the PD tube entry point. Application of 2-octylcyanoacrylate (Dermabond®, Ethicon, Inc., Somerville, NJ) around the exit site of the tube was effective in stopping this leak (Fig. 3).

## Conclusions

To our knowledge, this is the first report to describe the use of a percutaneous tube to successfully conduct PD for peritoneal drainage in an ELBW infant. This is the lowest-weight ELBW infant with successful PD reported to date.
